# Multiple Genomic Alterations, Including a Novel *AFF4::IRF1* Fusion Gene, in a Treatment-Refractory Blastic Plasmacytoid Dendritic-Cell Neoplasm: A Case Report and Literature Review

**DOI:** 10.3390/ijms25010305

**Published:** 2023-12-25

**Authors:** Yavuz Sahin, Y. Lynn Wang, Jianming Pei, Nashwa Mansoor, Michael Styler, Joseph R. Testa, Reza Nejati

**Affiliations:** 1 Department of Pathology, Fox Chase Cancer Center, Philadelphia, PA 19111, USA; yavuz.sahin@fccc.edu (Y.S.); yuelynn.wang@fccc.edu (Y.L.W.); jianming.pei@fccc.edu (J.P.); nashwa.mansoor@fccc.edu (N.M.); 2 Molecular Diagnostics Lab, Fox Chase Cancer Center, Philadelphia, PA 19111, USA; 3 Department of Bone Marrow Transplant and Cellular Therapies, Fox Chase Cancer Center, Philadelphia, PA 19111, USA; michael.styler@fccc.edu; 4 Clinical Cytogenomics Lab, Fox Chase Cancer Center, Philadelphia, PA 19111, USA; joseph.testa@fccc.edu; 5 Cancer Prevention and Control Program, Fox Chase Cancer Center, Philadelphia, PA 19111, USA

**Keywords:** BPDCN, blastic plasmacytoid dendritic-cell neoplasm, neoplasm, leukemia, plasmacytoid, Tagraxofusp

## Abstract

Blastic plasmacytoid dendritic cell neoplasm (BPDCN) is a rare hematologic malignancy with an aggressive clinical course and poor prognosis. The genetic abnormalities in BPDCN are heterogeneous; therefore, its molecular pathogenesis and the prognostic importance of genomic alterations associated with the disease are not well defined. Here we report a case of BPDCN with a novel AFF4::IRF1 fusion predicted to lead to a loss-of-function of the IRF1 tumor suppressor, somatic mutations of ASXL1, TET2, and MYD88, as well as multiple intrachromosomal deletions. The patient showed resistance to Tagraxofusp and Venetoclax, and he died about 16 months after diagnosis. Considering the predicted effect of the AFF4::IRF1 fusion on IRF1’s antitumor effects and immune regulation, and the possibility of its relevance to the aggressive course observed in this case, we propose further evaluation of the clinical significance of this fusion in BPDCN in future cooperative group studies and the consideration of therapeutic strategies aimed at restoring IRF1-dependent antineoplastic effects in such cases.

## 1. Introduction

Blastic plasmacytoid dendritic cell neoplasm (BPDCN) is a rare hematologic malignancy with an overall incidence estimated to be 0.04 per 100,000 people [1,2]. BPDCN is more common in older men with a 3:1 to 5:1 sex ratio and a median age of diagnosis of 60 to 70 years [3]. It has an aggressive clinical course and poor prognosis with a median overall survival of 2 years [2,4]. It is known to be derived from the precursors of plasmacytoid dendritic cells and mostly presents with skin lesions, with or without bone marrow or extramedullary involvement [2,5].

The fact that BPDCN is a rare disease and often presents with an unusual clinical progression and overlapping symptoms makes it a difficult disease to diagnose. A helpful hint in recalling its distinctive features involves the sequence “1234567”. BPDCN is distinguished by the expression of CD123+, CD4+, CD56+, and 70s (usually seen in older age) [2]. More than 50% of patients with BPDCN have chromosomal abnormalities, but a specific chromosomal change has not been demonstrated [6]. Most of the identified abnormalities have been deletions involving 5q, 12p, 13q13.1–14.2 (*RB1*), 6q, 15q, 9p21.3 (*CDKN2A/B*), and 7p12.2 (*IKZF1*). Deletions of 12p, encompassing the *ETV6* gene, are among the most common findings in BPDCN. Molecular analyses have identified recurrent driver mutations in *ASXL1*, *IDH1/2*, *IKZF1-3*, *NRAS*, *NPM1*, *TET1/2*, *TP53*, *U2AF1*, and *ZEB2* as well as rearrangements involving *MYB* [6,7,8,9]. Several transcription factors, e.g., *ETV6* and *TCF4*, have an important role in the proliferation of BPDCN cells [10,11]. Moreover, the B-cell lymphoma 2 (BCL2) protein plays a crucial role in BPDCN cell survival [12,13]. Recent advancements have revealed NF-κB pathway activation, disturbance in cholesterol balance, and breakdown of the TCF4-/BRD4-dependent transcription network in BPDCN. However, these alterations do not comprehensively uncover the regulatory mechanisms contributing to BPDCN development. Although several studies have explored the cytogenetics and molecular biology of BPDCN pathogenesis, neither have provided specific insights, leaving the use of genomic alterations for diagnosing BPDCN a significant challenge [14].

A better understanding of the pathophysiology of BPDCN has led to the development of new treatments. Today, various treatments are available for BPDCN including chemotherapeutics, autologous and allogeneic hematopoietic stem cell transplantations, Nuclear Factor-kappa B pathway inhibitors (Bortezomib), DNA hypomethylating agents (5-Azacytidine), BCL2 inhibitors (Venetoclax), bromodomain inhibitors, folate metabolism inhibitors (Pralatrexate) and CD123 targeted therapies (Tagraxofusp) [12,13,15]. However, despite the availability of treatment options, a majority of the patients have a poor prognosis and short survival times [4]. In addition to uncertainties in the pathophysiology and biology of BPDCN, the prognostic indicators remain unclear. Furthermore, the genetic abnormalities in BPDCN are heterogeneous; therefore, their prognostic significance is controversial [2].

Here we report a case of BPDCN with complex genomic abnormalities, various somatic mutations, and a novel *AFF4::IRF1* fusion. The patient showed resistance to Tagraxofusp (a fusion protein consisting of interleukin 3 or CD123 ligand fused to diphtheria toxin) and Venetoclax, that is possibly linked to the presence of this novel fusion. We also present a mini literature review of the genetic findings in BPDCN.

## 2. Case Presentation

The patient was an elderly man who presented with a painless dime-sized violaceous mass over his right anterior chest skin. A punch biopsy was performed on the lesion. The section of skin demonstrated a dense atypical cellular infiltrate extending from the upper dermis into the lateral and deep margins of the biopsy. The atypical cells were intermediate to large and oval to irregular in shape having open nuclear chromatin and one or more inconspicuous small nucleoli. Scattered mitotic figures were present. Immunostaining for CD3 and CD20 highlighted scattered interstitial and ill-defined collections of T-lymphocytes and only occasional interstitial B-lymphocytes which also form rare small collections. The atypical cell population was negative for CD3 and CD20 but strongly expressed CD4, CD123, CD56, and BCL2 with weak expression of BCL6 and CD68. The neoplastic cells were negative for CD34, MUM1, and lysozyme. At the time of diagnosis, bone marrow was not involved, and both cytogenetics and CMA studies [16] did not show any abnormalities. Ki67 showed a high proliferative index (approx. 70–80%). These findings confirmed the diagnosis of BPDCN [17].

**Figure 1:**
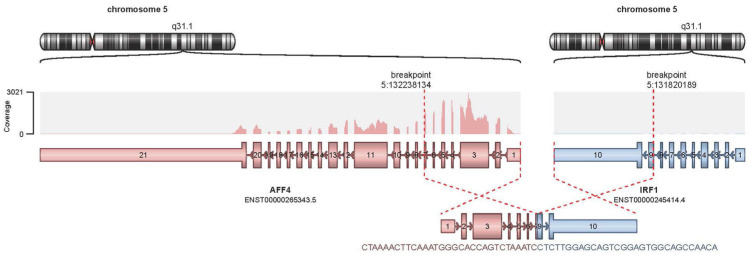
Display of *AFF4::IRF1* fusion and genes structures.

The patient showed complete resolution of skin nodules after initiating three doses of Tagraxofusp. The therapy was stopped due to a capillary leak, a typical side effect of Tagraxofusp. Four months later the patient developed local disease recurrence and Tagraxofusp was restarted. The patient received a total of 28 doses of Tagraxofusp, with good disease control. One year after the initial diagnosis, the peripheral counts at 1-week intervals revealed 18% and 35% blasts, respectively, suggesting bone marrow involvement, and a bone marrow biopsy was performed. Although the biopsy was suboptimal, flow cytometry of the bone marrow detected an abnormal cell population in the blast gate (40% of the total) with the following immunophenotype: CD4+, CD56+, CD123+, HLA-DR+, CD38 subset+, CD2+, CD3−, CD5−, CD7−, CD8−, CD45 dim+, CD34−, CD117−, MPO−, TdT−, CD13−, CD14−, CD16−, CD19−, CD20−, CD33−, and CD64. Lymphocytes accounted for about 21% of the total cells; they were small to intermediate in size. B cells (7% of lymphocytes) were polyclonal. T cells (81% of lymphocytes) had a CD4:CD8 ratio of 1:1, without an aberrant immunophenotype. Natural killer cells (CD56+/CD16+/CD3−) constituted 8% of lymphocytes. Large granular lymphocytes (CD57+/CD3+) represented 34% of lymphocytes. Myeloid populations showed decreased side scatter, consistent with hypogranularity. CD34+/CD117+ positive blasts were <1% of the total. The flow cytometry confirmed the presence of circulating BPDCN, indicating bone marrow involvement. The cytogenetic analysis reported a normal male karyotype. Analysis of bone marrow using a targeted Next Generation Sequencing (NGS) ribonucleic acid (RNA) fusion panel revealed an out-of-frame fusion between the *AFF4* and *IRF1* genes (Figure 1), which is predicted to be caused by a 417,945-bp deletion in chromosome 5 at band 5q31.1.

**Figure 2:**
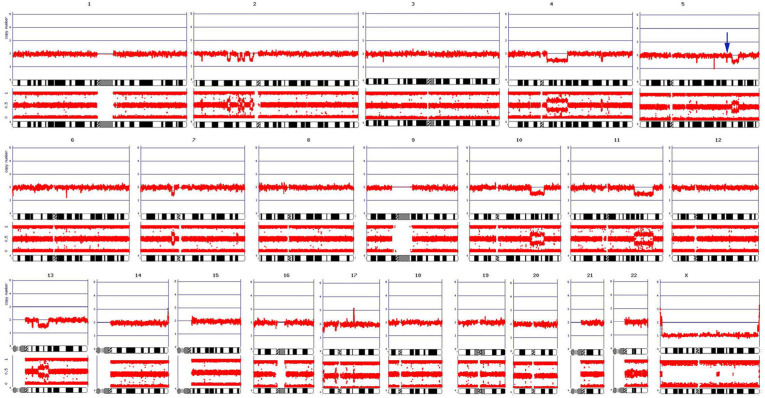
Chromosome microarray analysis (CMA). For each chromosome, the ordinate depicts the DNA copy number (not log2 ratio) in the upper panel and B-allele frequency (BAF) in the lower panel, loss of copy number is defined as less than two copies (diploid), and gain is defined as more than two copies; normally, BAF panels show three “bands”, representing all homozygous (upper and lower bands) and heterozygous (middle band) allele calls. Missing the middle band or an extra band in the middle indicates the loss of heterozygosity (LOH) or mosaicism. Note that chromosome coordinates are shown on the abscissa. The blue arrow above chromosome 5 indicates the focal deletion that gives rise to the *AFF4::IRF1* fusion shown in Figure 1.

Mutations in the *ASXL1*, *MYD88*, and *TET2* genes were detected via NGS of bone marrow DNA. According to the ClinVar Database, the *ASXL1* mutation (c.2077C>T) we observed results in a stop codon (p.Arg693*) that is a pathogenic/likely pathogenic variant reported previously in myelodysplastic syndrome and Bohring-Opitz syndrome. There were two novel *TET2* mutations. One of these had a single bp deletion, c.2551delC, which results in a frameshift (p.Pro851fs), and the other, c.2474C>G, results in a stop codon (p.Ser825*); all of these are expected to result in loss of function. The *MYD88* mutation is a 19-bp deletion that results in a frameshift, p.Ala6fs. The nomenclature defining these mutations is presented in Table 1. Chromosomal Microarray Analysis (CMA) revealed losses of segments in chromosome arms 2p, 4q, 5q, 7p, 10q, 11q, 13q, and 17p (specifically in p13.3) in a mosaic state representing about 50% of the cells (Figure 2). Notably, the deletions in 7p, 11q, and 13q encompass the tumor suppressor genes *IKZF1*, *ATM*, and *RB1*, respectively. In addition to the obvious deletions mentioned, a tiny deletion in chromosome 5 at band 5q31.1 was also observed; this focal deletion corresponds to the deletion that gave rise to the *AFF4::IRF1* fusion shown in Figure 1.

Flow cytometry of peripheral blood showed an abnormal CD4+, CD56+, CD123+ cell population (43% of total). CMA of a peripheral blood sample showed a similar profile to the previous bone marrow sample. Cytogenetic findings were again normal, suggesting that the genomically abnormal cells either did not divide in culture or that the abnormal metaphases did not spread sufficiently for karyotypic analysis. NGS sequencing of peripheral blood DNA revealed the same *TET2* and *ASXL1* mutations seen earlier in the bone marrow. However, the MYD88 mutation was not observed, and two new sequence variants were found, one in *KMT2C*, and the second, in *GNAS* (Table 2). However, the *GNAS* alteration (c.991G>A; p.Ala331Thr) is a variant of uncertain significance (VUS), and the *KMT2C* alteration (c.10_28del; Y366C) appears to be a VUS as well. Targeted RNA fusion analysis of the peripheral blood showed the same *AFF4::IRF1* fusion that was seen in the marrow. After developing leukemic transformation, treatment was then changed to Venetoclax until the patient relapsed and comfort care was initiated. A signed consent form was obtained from the patient and his legal heirs, which would allow the patient’s data to be presented as a case report. The patient passed away 16 months after initial diagnosis.

## 3. Discussion

In the current report, we present a case of BPDCN with a novel *AFF4::IRF1* fusion, accompanied by point mutations in several genes previously described in BPDCN (*ASXL1*, *TET2*). Mutations in genes related to DNA methylation pathways, such as TET2, often correlate with a poor prognosis in BPDCN [14]. Additionally, the CMA analysis revealed various chromosomal deletions leading to a loss of heterozygosity in several potentially critical tumor suppressor genes, including *IKZF1*, *ATM*, and *RB1*. These genomic alterations may have contributed to our patient’s unfavorable prognosis and resistance to Tagraxofusp and Venetoclax.

Like other AFF protein family members, AFF4 regulates gene transcription through elongation and chromatin remodeling. It is also thought to play a role in the differentiation of mesenchymal stem cells [18]. Depletion or amplification of the *AFF4* gene has been associated with various malignancies such as leukemia, head and neck squamous cell carcinoma, bladder cancer, and colorectal carcinoma [19,20,21]. *AFF4* is a common fusion partner in acute lymphoblastic leukemia/lymphoblastic lymphoma, chronic lymphocytic leukemia and adenocarcinoma of solid tumors, such as prostate, ovary, esophagus, and breast according to Mitelman’s Database (https://mitelmandatabase.isb-cgc.org) (accessed on 10 November 2023). Interferon regulatory factor (IRF) members show functional and specialized roles in the regulation of target gene expression. IRF1, a member of the IRF family, demonstrates functional diversity in the regulation of cellular response by activating the expression of a diverse set of target genes, depending on the cell type and the specific stimuli [22]. *IRF1* is a tumor suppressor gene and rearrangements of *IRF1* have a crucial role in the pathogenesis of some malignancies such as chronic myeloid leukemia and myelodysplasia [23,24]. As a tumor suppressor, it both suppresses tumor cell growth and stimulates immune response against tumor cells. Defects in this gene have been associated with gastric cancer, myelogenous leukemias, and lung cancer [25]. *IRF1*-associated fusions *IRF1::CEP20* and *IRF1::LPAR6* were documented in gastric adenocarcinoma and mature B-cell neoplasm (NOS), respectively, in the Mitelman Database. However, it remains unclear whether formation, progression, or response to the treatment of BPDCN is associated with AFF4- or IRF1-related abnormalities. To our knowledge, there is no prior report of an *AFF4::IRF1* fusion in BPDCN patients. In this context, this case report is the first to demonstrate an *AFF4::IRF1* fusion in a patient with BPDCN (Figure 1). Given that the *AFF4::IRF1* fusion is predicted to lead to IRF1 loss-of-function, this would adversely affect IRF1’s antitumor effects and immune regulatory function. If IRF1 alterations were found to be recurrent in BPDCN, consideration might be given to therapeutic strategies aimed at restoring IRF1-dependent antineoplastic effects for such cases. For example, restoration of IRF1-dependent anticancer effects has been achieved by MEK inhibition in human cancer cells, and IRF1 was found to play an essential role in apoptosis induced by Ras/MEK inhibition [26].

BPDCN is a rare and aggressive form of cancer. The absence of specific symptoms and the challenging nature of diagnosis often lead to late-stage detection, when the disease has already advanced throughout the body. Moreover, the incomplete understanding of the disease’s underlying pathological mechanisms and biological attributes compounds the diagnostic challenge, resulting in limited effective treatment choices [2]. Therefore, further research and treatment strategies, fostered by cancer research cooperative groups, are needed for this rare disease in order to improve the prognosis and survival of BPDCN patients. BPDCN is most frequently characterized by asymptomatic skin lesions seen in approximately 90% of patients at diagnosis, as in the present case. These lesions may present as solitary or multiple lesions, may spread widely, and range from caries-like lesions to plaques or nodules [2]. Bone marrow involvement, central nervous system infiltration, lymphadenopathy, splenomegaly, and/or cytopenias may accompany these features with varying severity [2]. In the current treatment-resistant case of BPDCN, our objective was to present all the genetic alterations we detected while highlighting a novel abnormality.

According to the 5th edition of the World Health Organization Classification of Haematolymphoid Tumors, BPDCN diagnosis requires expression of CD4, CD56, and CD123 and another pDC marker (CD123, TCL1, TCF4, CD304, and CD303) and the absence of lineage-associated antigens such as MPO, lysozyme, CD3, CD14, CD19, and CD34 [27,28]. There is no characteristic chromosome abnormality in BPDCN, but approximately 75% of these patients have complex karyotypic features [29]. The most common cytogenetic abnormalities include deletions of 5q, 6q, 9p (or monosomy 9), 12p, 13q, and 15q. Deletion of 12p is the most common finding in BPDCN [2]. NGS has enabled the search for gene mutations and altered expression of genes associated with BPDCN. Somatic mutations or over expression of *APC*, *ASXL1*, *ATM*, *BCL2*, *BCL6*, *BCL11A*, *BRAF*, *CDKN1B*, *CDKN2A*, *ETV6*, *GNAS*, *IDH1/2*, *IGLL1*, *IKZF1*, *KIT*, *KRAS*, *LRMP*, *MET*, *MLH1*, *MYC*, *NRAS*, *RB1*, *RET*, *SRSF2*, *TET2*, *TP53*, *U2AF1*, *VHL*, and *ZRSR2* have been reported in various cases of BPDCN [2,30,31,32,33,34]. However, many of these genes are also altered in myeloid neoplasms, so their specificity to support the diagnosis of BPDCN is only modest [2]. In addition, *MYC*, *MYB*, and *KMT2A* rearrangements have been reported in some studies of BPDCN [14,35,36]. In our case, deletions were detected in 2p, 4q, 5q, 7p, 10q, 11q, 13q, and 17p, and pathogenic mutations were detected in *ASXL1*, *TET2* (two), and *MYD88*, which are largely consistent with the literature.

*MYD88* is known as a cytoplasmic adapter protein involved in the antiviral response of blastic plasmacytoid dendritic cells [37]. A heterozygous mutation of the *MYD88* gene has been recently described in diffuse large B-cell lymphoma, splenic marginal zone lymphoma, and chronic lymphocytic leukemia [38,39,40]. While we identified a mutation in the *MYD88* gene in our case, Fandrino et al. tested nine fresh frozen skin biopsies of BPDCN for somatic mutations of *MYD88*, but found that none carried a mutation that affected the Toll/IL-1 receptor activity of MYD88 [37]. The results of another investigation [41] supported that study’s findings [37]. Thus, it seems that the relationship between *MYD88* and BPDCN is uncertain and requires further investigation.

Although CD123, a subunit of interleukin 3 receptor, is expressed in most CD34+ hematopoietic progenitors, its expression is maintained only in monocytic and granulocytic lineages [12]. The overexpression of CD123 was also demonstrated in some hematologic malignancies, particularly acute myeloid leukemia, hairy cell leukemia, and BPDCN [42]. However, positivity for CD4, CD45RA, CD56, and CD123, along with the absence of lineage-associated antigens is considered a pathognomonic phenotype of BPDCN [43]. Overexpression of CD123 is observed in 100% of patients with BPDCN [44]. Therefore, CD123 is one of the main targets in the current development of new therapies for myeloid malignancies [45]. Tagraxofusp is the first CD123-targeting agent approved for the treatment of BPDCN [46]. More than half of patients respond to Tagraxofusp therapy. While the rate of complete response is 70% in previously untreated patients over 70 years of age, the response rate is only 10% in previously treated patients >70 years of age (12). Another potential therapeutic agent is Venetoclax, a BCL2 inhibitor [13]. Although these two agents were used at different times in our patient, the individual died within approximately 16 months after diagnosis. The factors that have prognostic implications in the therapeutic response of BPDCN patients are mostly unknown [12]. In a retrospective study, age > 60 years at diagnosis, abnormal karyotype, and terminal deoxynucleotidyl transferase negativity were suggested to be associated with worse treatment responses [47]. In another study, multivariate Cox regression analysis revealed that advanced age, male gender, and BPDCN occurring as the first malignancy were independent prognostic factors for overall survival [48]. Yin et al. suggested that older age, multiple mutations, and mutations in the DNA methylation pathway were poor prognostic factors for BPDCN [49]. In an extended clinical-immunohistochemical study on a series of 91 well-documented BPDCN cases, it was shown that CD303 expression and high proliferative index based on Ki67 staining were significantly associated with longer survival [50]. Lucioni et al. showed that a 9p21.3 deletion is associated with a poor prognosis in BPDCN [51].

The novel *AFF4::IRF1* gene fusion and complex genetic karyotype potentially contributed to the patient’s poor clinical outcome. BPDCN cells express type I interferon. IRF1 (Interferon Regulatory Factor 1) regulates type I interferon secretion. IRF1 plays a crucial role in maintaining the delicate balance between self-renewal and differentiation of hematopoietic stem cells (HSCs). This tight regulation ensures a steady supply of blood cells throughout life, while also preventing uncontrolled cell growth. Therefore, the *AFF4::IRF1* gene may play a pivotal role for the pathogenesis of BPDCN. Therefore, exploring the potential correlation of this fusion with BPDCN pathogenesis through further studies and subsequently devising treatment alternatives could enhance the management of treatment-resistant BPDCN.

## 4. Conclusions

The genetic changes linked to BPDCN present a complex landscape, with diagnostic and prognostic implications that remain only partially understood. In a challenging treatment-resistant BPDCN case, we have detected a novel AFF4::IRF1 fusion. This fusion is expected to impair IRF1 function, affecting its tumor-suppressing and immune-regulatory roles. The identification of this novel fusion underscores the necessity for further exploration into its functional significance, potential role in BPDCN pathogenesis, and its implications for tailored treatment strategies. Understanding the clinical impact and therapeutic possibilities of IRF1 fusions in BPDCN requires additional comprehensive studies to pave the way for potentially targeted therapeutic interventions in BPDCN cases with an *AFF4::IRF1*.

## Figures and Tables

**Table 1:** Summary of the first NGS results from bone marrow.

Gene	Protein Change	cDNA Change	Allele Frequency	Reference
*MYD88*	p.Ala6fs	c.10_28delGACCGCGCTGAGGCTCCAG	27.5%	ENST00000396334
*TET2*	p.Ser825*	c.2474C>G	22.5%	ENST00000380013
*TET2*	p.Pro851fs	c.2551delC	22%	ENST00000380013
*ASXL1*	p.Arg693*	c.2077C>T	19%	ENST00000375687

**Table 2:** Summary of the second NGS results from peripheral blood.

Gene	Protein Change	cDNA Change	Allele Frequency	Reference
*KMT2C*	p.Tyr366Cys	c.1097A>G	6.4%	ENST00000262189
*TET2*	p.Ser825*	c.2474C>G	46.1%	ENST00000380013
*TET2*	p.Pro851fs	c.2551delC	44.0%	ENST00000380013
*ASXL1*	p.Arg693*	c.2077C>T	44.9%	ENST00000375687
*GNAS*	p.Ala331Thr	c.991G>A	47.0%	ENST00000371100

## Data Availability

All available data are included in the report.
